# Comparison of Overall and Domain-Specific Psychological Well-Being Between Homemakers and Employed Women: A Cross-Sectional Study From Perambalur District, Tamil Nadu

**DOI:** 10.7759/cureus.75103

**Published:** 2024-12-04

**Authors:** Vidhya Prasanthi Singaravel, Tamilarasan Muniyapillai, Karthikeyan Kulothungan, A Aswin

**Affiliations:** 1 Biochemistry, Government Medical College, Pudukottai, Pudukottai, IND; 2 Community Medicine, Dhanalakshmi Srinivasan Medical College and Hospital, Siruvachur, IND

**Keywords:** employment status, personal autonomy, psychological well-being, self-acceptance, socioeconomic factors, working women

## Abstract

Background

Women's psychological well-being (PWB) is influenced by various factors, including their occupational status and social roles. In India, where traditional and modern roles often intersect, understanding the PWB of homemakers and employed women is crucial for developing targeted mental health interventions. This study aimed to compare the overall and domain-specific PWB between homemakers and employed women in the Perambalur district, Tamil Nadu, using the 18-item Ryff's PWB Scale (PWBS).

Methodology

A cross-sectional study was conducted among 308 women (172 homemakers and 136 employed women) in the Perambalur district, Tamil Nadu, using convenience sampling. The 18-item Ryff's PWBS was used to assess PWB across six domains: autonomy, environmental mastery, personal growth, positive relations with others, purpose in life, and self-acceptance. Sociodemographic information was collected using a structured questionnaire. The internal consistency of the scale was assessed using Cronbach's alpha (α = 0.682, standardized α = 0.709). Data were analyzed using independent t-tests for overall scores and multivariate analysis of variance (MANOVA) for domain-specific comparisons.

Results

Homemakers demonstrated slightly higher overall PWB scores (69.35 ± 6.595) compared to employed women (68.21 ± 6.046), though this difference was not statistically significant (p = 0.121). MANOVA revealed significant multivariate effects of working status on PWB domains (Pillai's Trace = 0.071, p = 0.001, partial η^2^ = 0.071). Significant differences were found in self-acceptance (p = 0.001, partial η^2^ = 0.048), with homemakers scoring higher, and autonomy (p = 0.050, partial η^2^ = 0.012), where employed women scored higher. Sociodemographic analysis revealed that employed women were predominantly from urban areas (80, 75.5%) and higher socioeconomic classes (n = 31, 73.8% in Class 1 and n = 49, 69% in Class 2), while homemakers were more prevalent in rural areas (146, 72.3%) and lower socioeconomic classes.

Conclusion

The study reveals that occupational status influences specific domains of PWB rather than overall well-being scores. While homemakers demonstrated higher self-acceptance, employed women showed greater autonomy. The absence of significant differences in other domains suggests that both groups can achieve PWB through different pathways. These findings highlight the need for targeted mental health interventions that consider occupational status, socioeconomic factors, and geographical location when addressing women's PWB. Future public health initiatives should focus on enhancing domain-specific strengths while addressing potential vulnerabilities in both groups.

## Introduction

Psychological well-being (PWB) is a multidimensional construct that encompasses various aspects of positive functioning, including self-acceptance, personal growth, purpose in life, environmental mastery, autonomy, and positive relationships with others. [1,2] In recent years, there has been growing interest in understanding how different life roles and occupational status influence women's PWB, particularly in the context of evolving societal norms and expectations [3,4].

The comparison between housewives and working women's PWB has emerged as a significant area of research, especially in developing nations like India where traditional and modern roles often intersect [5,6]. Working women often navigate multiple roles, balancing professional responsibilities with domestic duties, which can impact their PWB in complex ways [4]. Studies have shown that employment can provide women with financial independence, social connections, and a sense of achievement, potentially enhancing their PWB [7]. However, the relationship between employment status and PWB is not straightforward, as working women may experience additional stressors related to role conflicts and work-life balance [8,9].

Conversely, housewives, who focus primarily on domestic responsibilities, may experience different challenges affecting their PWB [10]. Recent research has highlighted that both groups face unique stressors that can influence their mental health and overall life satisfaction [11]. A study conducted in North Bihar, India, found significant differences in PWB between housewives and working women, suggesting that occupational status may play a crucial role in determining mental health outcomes [5].

The COVID-19 pandemic has further complicated this dynamic, with studies indicating varying impacts on women's PWB based on their occupational status [12,13]. The lockdown periods particularly affected women's mental health, regardless of their working status, highlighting the need for continued research in this area [13]. Additionally, recent studies have emphasized the strong connection between PWB and physical health outcomes, suggesting that understanding these differences has important implications for overall health [14].

The measurement of PWB has evolved significantly since Ryff's pioneering work, which established the theoretical framework for understanding its multiple dimensions [1,2]. This multidimensional approach has become increasingly important in assessing the complex nature of well-being among different populations [15]. Recent studies in various parts of India have employed this framework to examine PWB among women, considering factors such as marital status, employment, and socioeconomic conditions [11,16].

Recent studies have also highlighted the importance of socioeconomic factors and regional variations in determining PWB among women [5,6]. The economic development and social changes in Tamil Nadu have created unique opportunities and challenges for both working women and housewives [4]. While some research has explored general well-being patterns, there is limited understanding of how specific domains of PWB might differ between these groups in semi-urban areas like Perambalur district [10]. Furthermore, the use of standardized assessment tools, particularly the Ryff's PWB Scale (PWBS), has proven valuable in capturing these nuanced differences across various populations [1,2]. Understanding these domain-specific variations could be crucial for developing targeted interventions and support systems for women in different occupational roles [11,14].

Despite the growing body of research in this area, there remains a need for region-specific studies that can account for local cultural and social contexts [4,6]. Tamil Nadu in India, with its unique social and cultural landscape, presents an important setting for examining these differences. Furthermore, while several studies have explored overall PWB, fewer have focused on domain-specific differences between homemakers and employed women [10,17]. Hence, this study aimed to compare the PWB among homemakers and employed women in Perambalur district, Tamil Nadu, India, by assessing both overall and domain-specific scores using the 18-item Ryff's PWBS (PWBS-18).

## Materials and methods

This was a community-based cross-sectional study, conducted in the field practice area of Dhanalakshmi Srinivasan Medical College and Hospital (DSMCH), a tertiary care teaching hospital in the Perambalur district of Tamil Nadu, India, between March 2023 and February 2024. The study protocol was approved by the Institutional Ethics Committee of DSMCH (approval number: IECHS/IRCHS/No. 392). Written informed consent was obtained from all participants after explaining the study objectives and procedures in their preferred language. Participant confidentiality was maintained throughout the study, and data were stored securely following standard research protocols.

Inclusion and exclusion criteria

The study included married women aged 18 years and above who were either homemakers or employed in any occupation. Employed women were defined as individuals who engaged in paid employment outside the home, whether in the formal or informal sectors, and met the specific criteria: (a) They must receive regular monetary compensation; (b) They must work at least 20 hours per week in economic activities outside the home. Homemakers included women who failed to meet these criteria. Women with diagnosed severe psychiatric illnesses, those unable to comprehend the questionnaire, and those unwilling to participate were excluded from the study.

Sample size estimation and sampling method

The sample size was calculated based on a previous study by Choudhary and Ahmed in India, which reported that 55% of working women demonstrated high levels of perceived PWB [5]. The minimum required samples in the study were calculated using Cochran's formula for calculating sample size: n = Z^2^ × p × (1-p) / d^2^, where p = 0.55 (prevalence) and d = 0.05 (5% absolute precision) at 95% confidence level (Z^2^ =3.84). The calculation yielded a minimum required sample size of 380 participants. The study population comprised adult women aged 18 years and above from both urban and rural areas within the field practice area of DSMCH. Participants were recruited using convenience sampling methodology.

Data collection procedure

An interviewer conducted face-to-face interviews with participants using a semi-structured questionnaire. The questionnaire included two main sections. The first section included sociodemographic information such as age, marital status, education, family type, residence, socioeconomic status, family size, spouse addiction status, occurrence of vital events, presence of chronic illness, and debt status. Socioeconomic status was classified based on the Modified BG Prasad classification [18]. The second section includes the Ryff's PWBS-18. The interviews were conducted in the local language, with appropriate translation and back-translation procedures followed to ensure content validity. Each interview lasted approximately 30-45 minutes, and privacy was maintained throughout the data collection process.

Study tool

PWB was assessed using Ryff's PWBS-18 [2]. This scale measures six dimensions of PWB: autonomy, environmental mastery, personal growth, positive relations with others, purpose in life, and self-acceptance [1]. Each subscale item is evaluated on a six-point Likert scale, with 1 representing "strongly disagree" and 6 indicating "strongly agree." The components for the Autonomy subscale are Q15, Q17, and Q18. The items on the Environmental Mastery subscale are Q4, Q8, and Q9. The items for the Personal Growth subscale are Q11, Q12, and Q14. The items for the Positive Relations with Others subscale are Q6, Q13, and Q16. The items for the Purpose in Life subscale are Q3, Q7, and Q10. The items for the Self-Acceptance subscale are Q1, Q2, and Q5.

Questions 1, 2, 3, 8, 9, 11, 12, 13, 17, and 18 need reverse scoring. Reverse-scored items are phrased contrary to the intended measurement of the scale. To compute subscale scores for each participant, aggregate the respondents' responses to the items of each subscale. Elevated scores indicate enhanced PWB across each category.

Table 1 shows the internal consistency reliability of the PWBS-18 was assessed using Cronbach's alpha (α = 0.682, standardized α = 0.709). While this reliability coefficient is slightly below the conventional threshold of 0.70, it is considered acceptable for a short-form version of an established scale [19,20]. Therefore, all items were retained for analysis to maintain the scale's validated structure and enable comparability with existing literature.

**Table 1:** Internal consistency reliability analysis of Ryff's PWBS-18 The analysis revealed a Cronbach's alpha of 0.682 (standardized α = 0.709), indicating questionable to acceptable internal consistency reliability. PWBS-18: 18-item Psychological Well-Being Scale

Item	Mean	Std. Deviation	Scale Mean if Item Deleted	Corrected Item-Total Correlation	Cronbach's Alpha if Item Deleted
Q1	6.44	0.753	87.63	0.396	0.668
Q2	5.13	1.732	88.94	0.253	0.671
Q3	6.12	1.31	87.95	0.281	0.669
Q4	4.09	2.024	89.98	0.292	0.667
Q5	4.61	1.961	89.46	0.337	0.661
Q6	4.99	1.879	89.08	0.208	0.677
Q7	4.3	2.253	89.78	0.068	0.7
Q8	5.67	1.43	88.4	0.131	0.683
Q9	6.28	0.82	87.79	0.372	0.668
Q10	4.95	2.12	89.12	0.239	0.675
Q11	5.51	1.56	88.56	0.372	0.659
Q12	5.81	1.327	88.26	0.498	0.65
Q13	5.55	1.389	88.53	0.169	0.679
Q14	4.8	2.076	89.27	0.475	0.641
Q15	3.4	1.974	90.67	0.294	0.667
Q16	5	1.983	89.07	0.358	0.658
Q17	5.69	1.381	88.38	0.13	0.683
Q18	5.72	1.475	88.35	0.255	0.671

Data analysis

The data analysis was performed using IBM SPSS Statistics for Windows, Version 26.0 (Released 2019; IBM Corp., Armonk, New York, United States). The descriptive statistics for the sociodemographic variables were presented as frequency and percentage for categorical variables, and for continuous variables it was presented as mean and SD. The overall PWB scores and individual domain scores were presented as mean, standard deviation, median, and IQR for both homemakers and employed women. To compare the overall PWB scores between homemakers and employed women, an independent t-test was conducted. To compare the domain-specific PWB scores between homemakers and employed women, the Mann-Whitney U Test was conducted. For comparing the six domains of PWB between the groups, a one-way multivariate analysis of variance (MANOVA) was performed. The assumptions of MANOVA, including multivariate normality and homogeneity of variance-covariance matrices, were checked using Box's M test. MANOVA showed significant differences (p < 0.05), and subsequent post-hoc analyses using Bonferroni correction were conducted to identify which specific domains differ between the groups.

## Results

**Figure 1:**
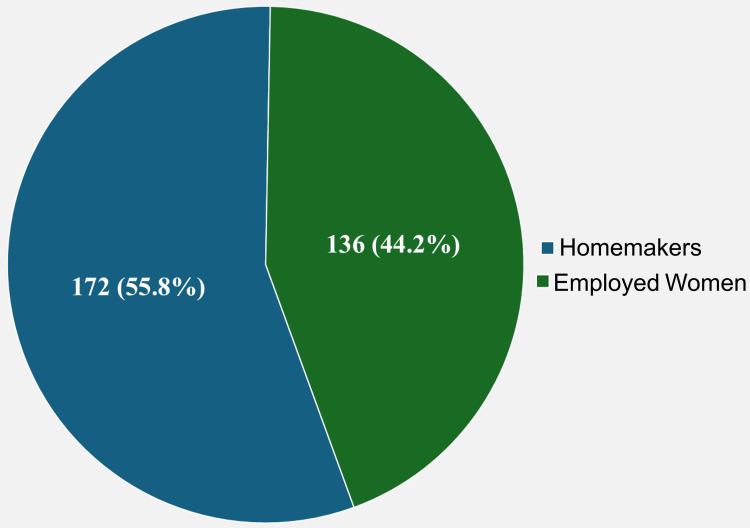
Distribution of study participants based on occupational status

Among the total participants (n= 308), 172 (55.8%) were homemakers, while 136 (44.2%) were employed women. Figure 1 illustrates the distribution of participants by their occupational status, showing a relatively balanced representation of both groups in the study population, with a slightly higher proportion of homemakers compared to employed women.

Table 2 shows the sociodemographic characteristics of study participants based on occupational status. The mean age of homemakers (44.7 ± 14.06 years) was higher than employed women (37.12 ± 9.96 years). Among married women, 143 (56.7%) were homemakers and 109 (43.3%) were employed women. For education status, most illiterate women were homemakers (n=21, 77.8%), while the majority of graduates were employed (n=84, 75%). In terms of family type, joint families had more homemakers (n=88, 71.5%), while nuclear families showed a more balanced distribution with 84 (46.2%) homemakers and 98 (53.8%) employed women. Rural residence was more common among homemakers (n=146, 72.3%), while urban residence was predominant among employed women (n=80, 75.5%). Regarding socioeconomic status, higher classes (Class 1 and 2) had more employed women (n=31, 73.8% and n=49, 69%, respectively), while lower classes (Class 4 and 5) had more homemakers (n=75, 78.1% and n=25, 92.6%, respectively). Family size distribution showed that medium-sized families (four to six members) were most common in both groups, with 104 (52.5%) homemakers and 94 (47.5%) employed women. Additional characteristics showed an equal distribution of spouse addiction between groups (n=36, 50% each). Vital events occurred in 21 (43.8%) homemakers and 27 (56.3%) employed women. Chronic illness was present in 33 (54.1%) homemakers and 28 (45.9%) employed women, while debts were reported by 82 (59%) homemakers and 57 (41%) employed women.

**Table 2:** Sociodemographic characteristics of study participants based on occupational status Data given as n (%) except for age, which is given as mean ± SD

Variable		Homemakers (n=172), n (%)	Employed women (n=136), n (%)
Age in years, mean ± SD		44.7 ± 14.06	37.12 ± 9.96
Marital status	Married	143 (56.7%)	109 (43.3%)
	Separated/ Divorced	6 (27.3)	16 (72.7)
	Widow	23 (67.6%)	11 (32.4%)
Education	Illiterate	21 (77.8%)	6 (22.2%)
	Primary school education	50 (80.6%)	12 (19.4%)
	High school education	73 (68.2%)	34 (31.8%)
	Graduated	28 (25%)	84 (75%)
Family type	Joint	88 (71.5%)	35 (28.5%)
	Nuclear	84 (46.2%)	98 (53.8%)
	Three generation	0 (0%)	3 (100%)
Residence	Rural	146 (72.3%)	56 (27.7%)
	Urban	26 (24.5%)	80 (75.5%)
Socioeconomic status	Class 1	11 (26.2%)	31 (73.8%)
	Class 2	22 (31%)	49 (69%)
	Class 3	39 (54.2%)	33 (45.8%)
	Class 4	75 (78.1%)	21 (21.9%)
	Class 5	25 (92.6%)	2 (7.4%)
Family size	Small size (<3)	50 (57.5%)	37 (42.5%)
	Medium size (4-6)	104 (52.5%)	94 (47.5%)
	Large size (>6)	18 (78.3%)	5 (21.7%)
Additional characteristics			
Addiction of husband		36 (50%)	36 (50%)
Vital events occurred		21 (43.8%)	27 (56.3%)
Presence of chronic illness		33 (54.1%)	28 (45.9%)
Presence of debts		82 (59%)	57 (41%)

Table 3 shows the comparison of overall and domain-specific PWB scores between homemakers and employed women. The overall PWB score was slightly higher among homemakers (mean: 69.35 ± 6.595) compared to employed women (mean: 68.21 ± 6.046). Among the six domains, autonomy scores were higher in employed women (mean: 13.44 ± 2.121; median: 14) compared to homemakers (mean: 12.88 ± 2.716; median: 14). Environmental mastery showed higher scores in homemakers (mean: 9.53 ± 3.879; median: 9) than employed women (mean: 8.88 ± 3.703; median: 9). Personal growth and positive relations with other domains showed slightly higher scores among homemakers (mean: 11.05 ± 2.423 and 12.2 ± 3.051, respectively) compared to employed women (mean: 10.65 ± 3.042 and 11.99 ± 3.607, respectively). Purpose in life was similar between groups, with employed women showing a marginally higher score (mean: 13.4 ± 2.715; median: 14) compared to homemakers (mean: 13.24 ± 2.207; median: 13.5). Self-acceptance was higher among homemakers (mean: 10.44 ± 1.488; median: 10) compared to employed women (mean: 9.86 ± 1.005; median: 10).

**Table 3:** Comparison of overall and domain-specific psychological well-being scores between homemakers and employed women IQR: interquartile range

Psychological well Being	Homemakers				Employed women			
	Mean	Std. Deviation	Median	IQR	Mean	Std. Deviation	Median	IQR
Overall score	69.35	6.595	69.5	65 - 74	68.21	6.046	68.5	64 - 72
Six Domains								
Autonomy	12.88	2.716	14	12 - 15	13.44	2.121	14	12.25 - 15
Environmental mastery	9.53	3.879	9	7 - 11	8.88	3.703	9	6 - 12
Personal growth	11.05	2.423	11	9 - 13	10.65	3.042	10	9 - 13
Positive relations with others	12.2	3.051	12	10 - 14	11.99	3.607	12	10 - 14
Purpose in life	13.24	2.207	13.5	12 - 15	13.4	2.715	14	11 - 15
Self-acceptance	10.44	1.488	10	9 - 11	9.86	1.005	10	9 - 10

Table 4 shows the comparison of overall PWB scores between homemakers and employed women. Homemakers demonstrated a marginally higher mean score (69.35 ± 6.60) compared to employed women (68.21 ± 6.05). Levene's test confirmed the homogeneity of variances (F = 1.410, p = 0.236). The independent t-test revealed no statistically significant difference in overall PWB scores between the groups (p = 0.121).

**Table 4:** Comparison of overall psychological well-being scores between homemakers and employed women Independent t-test was used; Equal variances assumed (Levene's test p = 0.236)

Group	N	Mean ± SD	Mean difference (95% CI)	t-statistic	p-value
Homemakers	172	69.35 ± 6.6	1.14 (-0.30, 2.57)	1.556	0.121
Employed women	136	68.21 ± 6.05			

**Figure 2:**
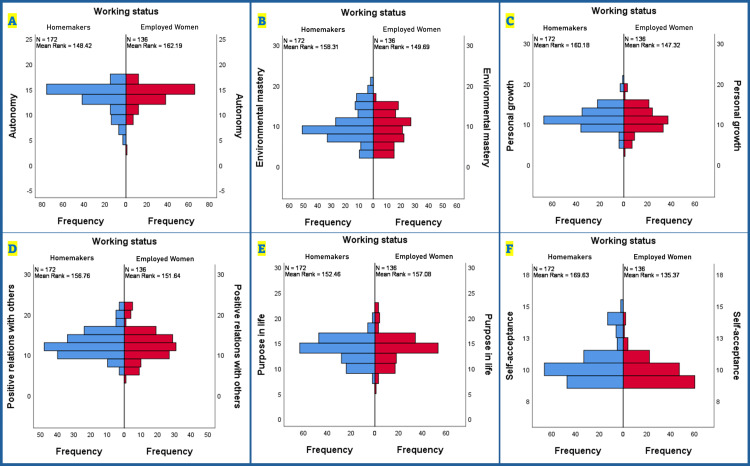
Distribution of domain-specific psychological well-being scores among homemakers and employed women

Figure 2 shows the comparison of the distribution of PWB domain scores between homemakers (n = 172, shown in blue) and employed women (n = 136, shown in red) using a Mann-Whitney U test. The autonomy domain (Figure 2A) demonstrated similar median scores between groups, though homemakers showed greater score variability. Environmental mastery (Figure 2B) exhibited a broader distribution pattern across both groups, indicating diverse levels of mastery over their environments. Personal growth scores (Figure 2C) revealed comparable patterns between the groups, with subtle differences in their distributions. The positive relations with other domains (Figure 2D) showed similar central tendencies between homemakers and employed women, suggesting comparable social relationship qualities. Purpose in life scores (Figure 2E) display relatively symmetrical distributions for both groups, indicating similar levels of life purpose perception. Finally, the self-acceptance domain (Figure 2F) showed more concentrated scores among employed women compared to a wider distribution among homemakers, suggesting more varied levels of self-acceptance in the latter group. The mean rank values are displayed for each group across all domains, providing quantitative measures of central tendency.

A Mann-Whitney U test was conducted to examine differences in PWB dimensions between employed women and homemakers (n = 308). The analysis revealed that five of the six dimensions showed no statistically significant differences between the two groups (Table 5). However, a significant difference was found in self-acceptance scores between employed women and homemakers (U = 9094.000, Z = -3.528, p <.001), indicating that employment status may influence individuals' level of self-acceptance.

**Table 5:** Mann-Whitney U Test results for psychological well-being dimensions *Statistically significant at p < 0.001

Dimensions	Mann-Whitney U	Z score	p-value
Autonomy	10650	-1.369	0.171
Environmental mastery	11041.5	-0.847	0.397
Personal growth	10719.5	-1.27	0.204
Positive relations with others	11307	-0.504	0.614
Purpose in life	11345.5	-0.458	0.647
Self-acceptance	9094	-3.528	0.000^*^

Table 6 presents the results of MANOVA comparing six domains of PWB between homemakers and employed women. Prior to the main analysis, Box's M test was conducted to examine the assumption of homogeneity of variance-covariance matrices. Box's M test indicated that the assumption of homogeneity of variance-covariance matrices was violated (M = 69.174, F = 3.22, p < 0.001). Given the relatively large and balanced sample sizes, we proceeded with MANOVA using Pillai's Trace as the test statistic due to its robustness to assumption violations. MANOVA revealed a significant multivariate effect of employment status on PWB domains (Pillai's Trace = 0.071, F (6, 301) = 3.827, p = 0.001, partial η² = 0.071). This result indicates that there are significant differences in PWB profiles between employed women and homemakers, though the effect size suggests these differences are modest in magnitude.

**Table 6:** Comparison of psychological well-being domains between homemakers and employed women: MANOVA results with univariate analyses ^*^p = 0.05 (marginally significant); ^**^p value < 0.001 was highly significant SE: standard error; CI: confidence interval; MANOVA: multivariate analysis of variance

Psychological Well-being Domains	Homemakers		Employed women		Mean difference (Homemakers—Employed women)	F-value	p-value	Partial η^2^
	Mean (SE)	95% CI	Mean (SE)	95% CI				
Autonomy	12.884 (0.188)	12.513, 13.255	13.441 (0.212)	13.024, 13.858	-0.557	3.864	0.05^*^	0.012
Environmental mastery	9.535 (0.290)	8.964, 10.105	8.875 (0.326)	8.233, 9.517	0.660	2.288	0.131	0.007
Personal growth	11.047 (0.207)	10.639, 11.454	10.647 (0.233)	10.189, 11.105	0.399	1.646	0.2	0.005
Positive relations with others	12.203 (0.252)	11.707, 12.700	11.985 (0.284)	11.427, 12.543	0.218	0.33	0.566	0.001
Purpose in life	13.238 (0.186)	12.872, 13.605	13.404 (0.210)	12.992, 13.817	-0.166	0.351	0.554	0.001
Self-acceptance	10.442 (0.099)	10.247, 10.636	9.860 (0.111)	9.641, 10.079	0.582	15.27	0.001^**^	0.048

Subsequent univariate analyses were conducted to examine differences between homemakers and employed women across six domains of PWB. The results revealed significant differences in two domains: self-acceptance and autonomy. Self-acceptance demonstrated the most robust difference between groups (p = 0.001, partial η² = 0.048), with homemakers reporting higher levels compared to employed women. The effect size indicates that group membership accounted for 4.8% of the variance in self-acceptance scores. Autonomy showed a marginally significant difference (p = 0.050, partial η² = 0.012), with employed women scoring higher than homemakers. The remaining four domains did not exhibit statistically significant differences between groups. The effect sizes for these non-significant domains were notably small, ranging from 0.001 to 0.007, suggesting minimal practical differences between the groups in these aspects of PWB.

## Discussion

The present study provides valuable insights into the PWB of homemakers and employed women in the Perambalur district, Tamil Nadu, revealing complex patterns in both overall well-being and specific psychological domains. The findings contribute to our understanding of how occupational status influences women's mental health in the Indian sociocultural context.

Our analysis revealed that homemakers demonstrated slightly higher overall PWB scores (69.35 ± 6.595) compared to employed women (68.21 ± 6.046), though this difference was not statistically significant (p = 0.121). This finding aligns with research by Choudhary and Ahmad, who reported similar patterns in their study of women in North Bihar [5]. The lack of significant differences in overall scores suggests that employment status alone may not be the primary determinant of women's PWB, supporting Sinha's assertion that multiple roles and their successful management are more crucial for psychological health than employment status per se [4].

The multivariate analysis revealed significant differences in PWB profiles between the groups (Pillai's Trace = 0.071, p = 0.001), particularly in the self-acceptance and autonomy domains. The higher self-acceptance scores among homemakers (p = 0.001, partial η² = 0.048) suggest that these women may have developed a stronger sense of identity and satisfaction within their chosen role. This finding resonates with research by Chaudhry and Chhajer, who emphasized the importance of role satisfaction in PWB [21].

Employed women demonstrated marginally higher autonomy scores (p = 0.050, partial η² = 0.012), consistent with findings by De-Juanas et al. regarding the relationship between autonomy and PWB [22]. This increased autonomy might be attributed to financial independence and workplace empowerment, as suggested by Houston et al. in their comparative study of employed women and homemakers [8].

The sociodemographic analysis revealed important patterns that may influence PWB. The higher prevalence of homemakers in rural areas (146,72.3%) and employed women in urban areas (80, 75.5%) reflects broader sociocultural and economic factors affecting women's employment choices. This geographic distribution pattern aligns with findings by Maeda et al., who identified significant associations between domestic work stress and psychological health outcomes across different residential settings [9].

The concentration of employed women in higher socioeconomic classes (n = 31, 73.8% in Class 1 and n = 49, 69% in Class 2) suggests that economic status and educational attainment significantly influence women's occupational choices. This observation supports research by Bramhankar et al., who found strong correlations between socioeconomic status and life satisfaction among Indian adults [23].

The absence of significant differences in environmental mastery, personal growth, positive relations, and purpose in life between groups is particularly noteworthy. This finding suggests that both homemakers and employed women can achieve similar levels of fulfillment in these domains through different pathways, supporting Ryff and Keyes' conceptualization of PWB as a multidimensional construct [2].

Recent research by Beulah suggests that the relationship between employment status and PWB is mediated by various factors, including social support, family dynamics, and personal aspirations [17]. This may explain why both groups showed comparable scores in several domains despite different daily experiences and challenges.

The similar scores in positive relations across both groups indicate that women can develop and maintain meaningful social connections regardless of their occupational status. This finding aligns with research by Arcos-Romero and Calvillo, who emphasized the importance of social relationships in women's overall well-being, regardless of their professional status [24].

The marginally higher environmental mastery scores among homemakers (mean: 9.53 ± 3.879) compared to employed women (mean: 8.88 ± 3.703) suggest that managing household responsibilities may enhance women's sense of control over their immediate environment. This finding corresponds with research by Parmar, who found that homemakers often develop strong organizational and management skills through their domestic responsibilities [25].

The slightly higher personal growth scores among homemakers challenge the common assumption that professional employment is necessary for personal development. This finding supports research by Chaurasia and Kumari, who found that both employed and non-employed women can experience significant personal growth through different life experiences and responsibilities [6].

The socioeconomic distribution pattern in our study, with employed women predominantly in higher classes, raises important questions about access to employment opportunities and its relationship with PWB. This observation aligns with findings by Dhanabhakyam and Sarath, who identified socioeconomic status as a significant moderator of PWB among women [15].

The findings of this study have significant implications for developing targeted mental health interventions and support systems for women across different occupational statuses. The identification of distinct PWB profiles between homemakers and employed women suggests the need for tailored mental health promotion strategies [14]. For instance, the higher autonomy scores among employed women indicate that workplace mental health programs should focus on maintaining and enhancing this strength while addressing potential challenges in self-acceptance. Conversely, community-based mental health initiatives for homemakers could focus on enhancing autonomy while building upon their stronger self-acceptance [3].

The socioeconomic disparities observed in our study, particularly the concentration of employed women in higher socioeconomic classes, highlight the need for public health policies that address barriers to employment and mental health support for women in lower socioeconomic groups [26]. Additionally, the similar scores in positive relations and purpose in life across both groups suggest that community-based support networks and social engagement programs could be equally effective for both homemakers and employed women, potentially serving as a cost-effective public health intervention strategy. Healthcare providers and public health practitioners should consider these findings when designing preventive mental health services and wellness programs, ensuring that interventions are culturally sensitive and address the specific psychological needs of both homemakers and employed women in different socioeconomic contexts [12].

Strengths of the study

The study demonstrated several notable strengths in its approach and execution. The research utilized the validated Ryff's PWBS-18, providing a comprehensive assessment of both overall and domain-specific well-being. The methodology was strengthened by relatively balanced sample sizes between groups and the application of appropriate statistical analyses, including MANOVA and Mann-Whitney U tests. The study's robustness was further enhanced by using Pillai's Trace to address violated assumptions in statistical analysis. Additionally, the research incorporated a detailed examination of socio-demographic variables, including various family-related factors such as family type, size, and spouse addiction, as well as health and financial considerations like chronic illness and debts. This comprehensive approach provided a rich context for understanding the relationship between occupational status and PWB.

Limitations

Several limitations should be considered when interpreting the study's findings. The cross-sectional design inherently limits causal inference, and the focus on a single geographic location (Perambalur district) may affect the generalizability of results to other populations. The study's measurement approach faced some challenges, including relatively low internal consistency (Cronbach's α = 0.682) and the use of a shortened version of the PWBS, which might not capture the full complexity of the PWB construct. There were also analytical limitations, such as the violation of the homogeneity assumption in MANOVA and limited control for potential confounding variables. The absence of analysis regarding interaction effects between demographic variables represents another limitation of the study's scope.

## Conclusions

This study provides valuable insights into the complex relationship between women's occupational status and PWB in the Perambalur district of Tamil Nadu. While the overall PWB scores were comparable between both groups, the study revealed significant domain-specific differences. Homemakers demonstrated higher self-acceptance, suggesting a more stable self-concept and greater acceptance of their life choices, while employed women showed higher autonomy, reflecting increased independence and decision-making opportunities. The absence of significant differences in environmental mastery, personal growth, positive relations, and purpose in life domains indicates that both groups find distinct ways to fulfill these aspects of PWB. These findings challenge common assumptions about the relative advantages of employment versus staying at home and suggest that PWB is more nuanced than previously understood, influenced by factors such as family support, personal choice, and social circumstances.

Future research should prioritize longitudinal studies across diverse geographical and socioeconomic contexts to better understand the temporal dynamics of PWB among women. Healthcare providers and policymakers should develop targeted mental health interventions addressing the specific needs of both homemakers and employed women while implementing community support programs that validate women's occupational choices. Establishing support groups and counseling services, along with educational initiatives focusing on coping strategies, would benefit both groups. Workplace policies should be designed to promote work-life balance, while community awareness programs should focus on destigmatizing and supporting women's choices regarding employment and domestic roles.

## References

[1] Ryff CD (1989). Happiness is everything, or is it? Explorations on the meaning of psychological well-being. J Pers Soc Psychol.

[2] Ryff CD, Keyes CLM (1995). The structure of psychological well-being revisited. J Pers Soc Psychol.

[3] Matud MP, López-Curbelo M, Fortes D (2019). Gender and psychological well-being. Int J Environ Res Public Health.

[4] Sinha S (2017). Multiple roles of working women and psychological well-being. Ind Psychiatry J.

[5] Choudhary DL, Ahmad DA (2017). A study of psychological well-being among housewives and working women of Mithila region, North Bihar, India. Indust Res.

[6] Chaurasia DK, Kumari a (2023). A study of psychological well-being and general self-efficacy among housewives and working women. Int Educ Res J.

[7] Warr P, Parry G (1982). Paid employment and women's psychological well-being. Psychol Bull.

[8] Houston BK, Cates DS, Kelly KE (1992). Job stress, psychosocial strain, and physical health problems in women employed full-time outside the home and homemakers. Women Health.

[9] Maeda E, Nomura K, Hiraike O, Sugimori H, Kinoshita A, Osuga Y (2019). Domestic work stress and self-rated psychological health among women: a cross-sectional study in Japan. Environ Health Prev Med.

[10] Parihar N, Agarwal U (2015). Study of psychological well-being in working and non-working menopausal women. Indian J Health Wellbeing.

[11] Yadav V, Yadav N, Sharma S (2023). The relationship between perceived stress and psychological well-being among working women and housewives. Int J Indian Psychol.

[12] Sharma D, Barla MA, Munjal Y, Roushan R (2021). A survey on mental wellbeing among Indian population during lockdown-2 of COVID-19 pandemic. Epidem Int.

[13] Patel JK, Parmar N (2021). The psychological well-being of working women during Covid-19 pandemic in India: a web based survey. Indian J Physiother Occup Ther.

[14] Hernandez R, Bassett SM, Boughton SW, Schuette SA, Shiu EW, Moskowitz JT (2018). Psychological well-being and physical health: associations, mechanisms, and future directions. Emot Rev.

[15] Dhanabhakyam M, Sarath M (2023). Psychological wellbeing: a systematic literature review. Int J Adv Res Sci Comm Tech.

[16] Gupta G, Nafis N (2014). Does marital adjustment and psychological well-being differences in working and non-working female?. Int J Indian Psychol.

[17] Beulah J, Prathap G, Vinothina Vinothina (2021). Study on psychological well-being of working women. Ann Rom Soc Cell Biol.

[18] Mohanty M, Patle RA, Narlawar UW (2024). Modified BG Prasad and modified Kuppuswami socio-economic status scales: revision and updates for January 2024. Prev Med Res Rev.

[19] Hair JF, Black WC, Babin BJ, Anderson RE (2019). Multivariate Data Analysis. 8th ed. : Multivariate Data Analysis. 8th ed.

[20] Taber KS (2018). The use of Cronbach's alpha when developing and reporting research instruments in science education. Res Sci Educ.

[21] Chaudhry S, Chhajer R (2023). Enhancing psychological well-being of school teachers in India: role of energy management, thriving, and stress. Front Psychol.

[22] De-Juanas Á, Bernal Romero T, Goig R (2020). The relationship between psychological well-being and autonomy in young people according to age. Front Psychol.

[23] Bramhankar M, Kundu S, Pandey M, Mishra NL, Adarsh A (2023). An assessment of self-rated life satisfaction and its correlates with physical, mental and social health status among older adults in India. Sci Rep.

[24] Arcos-Romero AI, Calvillo C (2023). Sexual health and psychological well-being of women: a systematic review. Healthcare (Basel).

[25] Parmar R (2018). Psychological well-being of working and non-working women. Res Guru Online J Multidiscip Subj.

[26] Morales-Rodríguez FM, Espigares-López I, Brown T, Pérez-Mármol JM (2020). The relationship between psychological well-being and psychosocial factors in university students. Int J Environ Res Public Health.

